# Carta ao editor sobre “Sistematização das etapas para impressão de modelos 3D de deformidades ortopédicas”

**DOI:** 10.1055/s-0045-1811685

**Published:** 2025-09-08

**Authors:** Amnuay Kleebayooon, Viroj Wiwanitkit

**Affiliations:** 1 Consultor acadêmico particular, Samraong, Oddar Meanchey, Camboja.; 2 Saveetha Medical College and Hospitals, Saveetha Institute of Medical and Technical Sciences, Thandaram, Kancheepuram District, Tamil Nadu, Índia.

Caro Editor, “Sistematização das etapas para impressão de modelos 3D de deformidades ortopédicas” 1 é um artigo interessante. O estudo descreveu um método de criação de modelos ósseos e articulares específicos do paciente utilizando impressão tridimensional (3D) e tomografias computadorizadas (TCs) pré-operatórias para gerar um modelo anatômico digital. O método envolve segmentação de dados com base na Comunicação de Imagens Digitais em Medicina (Digital Imaging and Communications in Medicine, DICOM, em inglês) em programa de imagem 3D, traduzindo-os para STL para posterior impressão com tecnologia de modelagem por deposição fundida ( *fused deposition modeling* , FDM, em inglês). Este método permite melhor planejamento cirúrgico e personalização da cirurgia ortopédica.

Embora o estudo forneça uma base para a impressão 3D em cirurgia ortopédica, ele mostra alguns desafios técnicos. Primeiro, a previsão de conversão variou com base na qualidade da imagem original e na técnica de segmentação. Erros nessas fases podem resultar em imprecisões no modelo impresso final, influenciando o desfecho cirúrgico. Além disso, o estudo não abordou a escolha de materiais para impressão 3D, que é crítica para as qualidades mecânicas do modelo impresso, particularmente quando são destinados ao uso pré-operatório para exame de elos anatômicos complicados. Os materiais impressos diferem em resistência, flexibilidade e biocompatibilidade e devem ser analisados minuciosamente quanto à estabilidade.

Dada a pesquisa existente e a metodologia proposta, os componentes emocionais e psicológicos do planejamento pré-operatório com modelos impressos em 3D são frequentemente desconsiderados. Modelos personalizados podem fornecer retorno tátil e visual para cirurgiões e pacientes, melhorando a compreensão e a comunicação sobre o procedimento cirúrgico e diminuindo a ansiedade. Além disso, embora o artigo descreva o processo de impressão, não aborda de maneira adequada a natureza iterativa da personalização do modelo, o que pode implicar a montagem de uma equipe multidisciplinar de cirurgiões, radiologistas e engenheiros biomédicos para otimizar o modelo com base nos resultados de simulações cirúrgicas, aumentando a precisão e a utilidade do modelo impresso.

Os hospitais devem estabelecer protocolos que incorporem o processo de impressão e a colaboração multidisciplinar. Adotar uma abordagem interdisciplinar pode impulsionar a inovação do *design* do modelo, fornecendo avaliações de diferentes pontos de vista, o que pode melhorar a precisão e a usabilidade do modelo no centro cirúrgico. Além disso, a equipe cirúrgica deve ser treinada não apenas na tecnologia, mas também em sua utilização em contatos e comunicação com pacientes.

Para aprimorar a aplicação terapêutica da tecnologia de impressão 3D, os programas educacionais devem incluir oficinas práticas que abranjam os elementos técnicos e clínicos da manufatura aditiva, assim como todo o fluxo de trabalho, permitindo que os médicos ortopedistas adaptem a tecnologia às suas necessidades. Usando técnicas práticas, os cirurgiões podem experimentar vários designs e parâmetros de impressão, gerando um ambiente criativo que pode levar a novas soluções para desafios cirúrgicos. Pesquisas futuras podem, também, explorar a fusão da impressão 3D com realidade aumentada (RA) ou realidade virtual (RV).

Essas tecnologias podem permitir que os cirurgiões estudem modelos 3D no espaço virtual antes da cirurgia, ajudando-os a entender melhor os intrincados elos anatômicos espaciais. Explorar essa intersecção pode levar a avanços significativos na medicina personalizada, que retrataria precisamente a anatomia única de cada paciente, resultando em melhores desfechos cirúrgicos. Além disso, desenvolver de algoritmos adaptativos capazes de refinar automaticamente os modelos com base no retorno do cirurgião e nos desfechos pós-operatórios pode criar um ciclo fechado que aumenta continuamente a precisão do modelo, levando a mudanças da prática ortopédica.
